# Association of GT microsatellite polymorphism in TLR 2 gene with leprosy

**DOI:** 10.1186/1471-2334-12-S1-P12

**Published:** 2012-05-04

**Authors:** NC Suryadevara, NV Sanjeev, Vijaya Lakshmi Valluri, Suman Jain, MPJS Anandraj

**Affiliations:** 1 LEPRA India – Blue Peter Public Health & Research Centre, Cherlapally, Hyderabad, India; 2 Thalasemmia and Sickle Cell Anaemia Society TSCS, Hyderabad, India

## Background

Toll-like receptor 2 (TLR2) is critical in bringing up immune responses to mycobacterial infections. The mutations in TLR2 are known to confer susceptibility for severe infection with mycobacteria. TLR2 may diminish response to mycobacterial proteins and place individuals at risk of developing leprosy. We investigated the association of GT repeat polymorphism in intron2 of TLR2 gene with leprosy in south Indian patients.

## Methods

A total of 20 leprosy patients and 45 contacts were enrolled in the study. Primers were designed using Primer3 software for PCR amplification of the TLR2 gene. The number of GT repeats was confirmed by sequencing. Two-tailed Chi-Square test was performed to check the association. p less than 0.05 was considered to be statistically significant.

## Results

The number of GT repeats varied from 13 to 24 in both the groups studied. The frequency of patients with (GT) 13 repeats was significantly low (p=0.04, OR=0.318) and that of (GT) 14 repeats (p=0.04, OR=7.76) was significantly high.

## Conclusion

Our results suggest that an individual with (GT) 13 repeats may be resistant and those with (GT) 14 repeats may be susceptible to leprosy. Furthermore, elucidation of functional relevance studies such as gene expression and proteomics may reveal the influence and role of these repeat number variations in leprosy.

